# School health promotion and the consumption of water and sugar-sweetened beverages in secondary schools: a cross-sectional multilevel study

**DOI:** 10.1186/s12889-023-16123-7

**Published:** 2023-07-05

**Authors:** Lisanne Vonk, Iris Eekhout, Tim Huijts, Mark Levels, Maria W. J. Jansen

**Affiliations:** 1 Academic Collaborative Center for Public Health Limburg, Public Health Service South Limburg, P.O. Box 33, 6400 AA Heerlen, The Netherlands; 2 Department of Health Services Research, Care and Public Health Research Institute (CAPHRI), Maastricht University, P.O. Box 616, 6200 MD Maastricht, The Netherlands; 3 Expertise Center Child Health, Netherlands Organisation for Applied Scientific Research (TNO), P.O. Box 3005, 2301 DA Leiden, The Netherlands; 4 Research Centre for Education and the Labour Market (ROA), School of Business and Economics, Maastricht University, Postbus 616, 6200 MD Maastricht, The Netherlands; 5 Centre for Global Health Inequalities Research (CHAIN), Department of Sociology and Political Science, Norwegian University of Science and Technology (NTNU), P.O. Box 8900, NO-7491 Trondheim, Norway

**Keywords:** School health promotion, Multilevel analysis, Secondary school, Water consumption, SSB consumption

## Abstract

**Background:**

Overweight among adolescents remains a serious concern worldwide and can have major health consequences in later life, such as cardiovascular diseases and cancer. Still, 33% of secondary school adolescents in the Netherlands consume sugar-sweetened beverages daily and over 26% do not consume water every day. The Dutch Healthy School program was developed to support schools in stimulating healthier lifestyles by focusing on health education, school environments, identifying students’ health problems, and school policy. We examined the variation between secondary schools regarding the daily consumption of water and sugar-sweetened beverages and whether this variation can be explained by differences between schools regarding Healthy School certification, general school characteristics, and the school population.

**Methods:**

We performed a cross-sectional multilevel study. We used data from the national Youth Health Monitor of 2019 on secondary schools (grades 8 and 10, age range about 12 to 18 years) of seven Public Health Services and combined these with information regarding Healthy School certification and general school- and school population characteristics. Our outcomes were daily consumption of water and sugar-sweetened beverages. In total, data from 51,901 adolescents from 191 schools were analysed. We calculated the intraclass correlation to examine the variation between schools regarding our outcomes. Thereafter, we examined whether we could explain this variation by the included characteristics.

**Results:**

The school-level explained 4.53% of the variation in the consumption of water and 2.33% of the variation in the consumption of sugar-sweetened beverages. This small variation in water and sugar-sweetened consumption could not be explained by Healthy School certification, yet some general school- and school population characteristics did: the proportion of the school population with at least one parent with high educational attainment, the educational track of the adolescents, urbanicity (only for water consumption) and school type (only for sugar-sweetened beverages consumption).

**Conclusions:**

The low percentages of explained variation indicate that school-level characteristics in general (including Healthy School certification) do not matter substantially for the daily consumption of water and sugar-sweetened beverages. Future research should examine whether school health promotion can contribute to healthier lifestyles, and if so, under which level of implementation and school conditions.

**Supplementary Information:**

The online version contains supplementary material available at 10.1186/s12889-023-16123-7.

## Background

Overweight among adolescents remains a serious concern worldwide [1]. This is alarming since childhood overweight regularly tracks into adulthood [2] and can have major health consequences such as type 2 diabetes, cardiovascular diseases, and cancer [3]. Additionally, previous research has shown that overweight is also associated with decreased mental health in adolescents [4]. Having unhealthy dietary behaviours is one of the most important causes of overweight in adolescents [5]. One of these behaviours is the consumption of sugar-sweetened beverages (SSBs). Since SSBs provide minimal to no satiety, people are at risk of overconsumption [6, 7]. This can lead to weight gain since SSBs are one of the main sources of added sugars, but have little nutritional value [8, 9]. It is therefore highly preferable from a health perspective to substitute the consumption of SSBs for water consumption, as water contains no sugar and calories [10].

To stimulate healthier behaviours among children and adolescents worldwide, the World Health Organization (WHO) developed a whole-school approach called the Health Promoting Schools (HPS) framework [11, 12]. A whole-school approach focusses on different aspects of the school context, such as the environment, healthy school policy, health education, involving the community and collaboration with regional health services [13]. The school setting is important for health promotion in the western world, since it is an effective way to reach almost all children and adolescents due to compulsory schooling [11]. The Dutch Healthy School (HS) program, one of the variants of school health promotion (SHP) in the Netherlands, is to a great extent in line with this HPS framework and is supported by the Dutch government [14]. The HS program aims to support schools in stimulating healthier lifestyles among primary-, secondary- and secondary vocational school students [14]. This program is necessary since the above-mentioned unhealthy behaviours are also an issue in the Netherlands. According to the results of the Dutch national Youth Health Monitor of 2019, 33% of secondary school adolescents that participated in the questionnaire consumed SSBs daily and over 26% of adolescents did not consume water every day [15]. To obtain an HS program certificate, a school has to acquire a topic certificate for a health theme, such as nutrition, and fulfill minimum requirements. These requirements are related to four pillars: health education, school environments (both physical and social), identifying students who need extra attention or referral and healthy school policy [14]. In 2019–2020, topic certificates were linked to five health topics for secondary schools: nutrition, physical activity, well-being, smoking, alcohol and drug prevention and relationships and sexuality. To acquire the nutrition certificate, there are requirements such as implementing nutrition-promoting interventions related to the four pillars (e.g. the HS Canteen, a nutrition policy, and approved educational activities) [16].

Even though many schools have implemented the HS program, little is known about the degree of implementation or its impact. However, results of a review regarding the implementation of the HPS framework indicated that schools focus more on their curriculum, and ethos and environment, than on involving families or the community [17]. Previous studies on the impact of the HS program typically focused on health education while neglecting the other three pillars [18]. A review by Wang and Stewart [19] that focused on nutrition-promoting interventions found evidence that school-based interventions can reduce the consumption of SSBs and increase the consumption of water, but other evidence is inconclusive or is only found for subgroups [20–22]. We can shed more light on these seemingly contradicting results if we take into account the school context, e.g. general school- and school population characteristics. For example, socioeconomic characteristics of parents, such as their educational attainment and income, are associated with the health of their children [23]. Other possible moderators are urbanicity of the school area due to the proximity of supermarkets [24, 25] or having a migration background due to cultural differences with regard to dietary behaviours [26]. To better understand the impact of the HS program on the consumption of water and SSBs, general school- and school population characteristics should be taken into account.

Since the HS program is implemented at the school-level, we hypothesise that the program will explain variation between schools in dietary intake on the individual-level. The current paper contributes to the existing literature by answering the following research question: To what extent can the variation between secondary schools in the Netherlands regarding the daily consumption of water and SSBs by adolescents be explained by differences between schools regarding HS certification, general school characteristics, and the school population? We also examined whether these general school- and school population characteristics moderated the association between HS certification and the daily consumption of water and SSBs since we hypothesised that its impact could differ in different school contexts.

## Methods

### Study design and study population

This study is part of a larger evaluation study of the Dutch HS program [27] to discover the implementation conditions under which the program has an impact on lifestyle, health, and learning outcomes. The design of the current sub-study was a cross-sectional multilevel study that took place in the Netherlands. Our study population consisted of adolescents from secondary schools located in 7 out of the 25 Public Health Services that participated in the national Youth Health Monitor of 2019 [28]. The Youth Health Monitor is a national survey that is repeated at least every four years and the aim is that all secondary schools participate, although participation is voluntarily. Adolescents in year 2 and year 4 (ISCED-2 and ISCED-3) of secondary school, equivalent to grades 8 and 10, fill out the survey anonymously during school hours. The outcomes used in this study, the self-reported daily consumption of water and SSBs, and other individual characteristics were standard questions in the Youth Health Monitor. All surveys were conducted from September until December in 2019, i.e. prior to the Covid-19 pandemic. The Association of Regional Public Health Services (*GGD GHOR*) provided data regarding HS certification and whether schools received more intensive support in using the HS program in the promotion of a specific health topic in the school years 2015–2016 to 2019–2020. Data regarding other general school- and school population characteristics were obtained from the *Netherlands Cohort Study on Education* (NCO), which was launched by the Netherlands Initiative for Education Research (NRO). The NCO contains data of all publicly funded schools in the Netherlands [29], i.e. almost all schools, since there are very few privately funded schools in the Netherlands. All data were combined using an encrypted school identifier. Where location identifiers of schools were missing and we could match only one school location in the NCO dataset, we assumed all data corresponded to that school location.

We did not include adolescents with no school identifier or location code. We also excluded special needs schools, duplicated schools, and schools with multiple school identifiers. Lastly, adolescents outside of grades 8 and 10, adolescents who indicated they were not following pre-vocational secondary education (vmbo), senior general secondary education (havo) or pre-university education (vwo) but a different educational track, adolescents from schools that could not be identified in the NCO dataset, and adolescents with no individual data were also excluded.

### Measurements

#### Consumption of water and SSBs

The study outcomes were analysed at the student-level and operationalised with a dummy variable indicating whether an adolescent consumed at least one glass of *water* every day, and one dummy indicating whether the adolescent consumed *SSBs* every day. The standard survey of the Youth Health Monitor did not measure the consumed quantity of water and SSBs per day. SSBs were specified as soda, energy drinks, sport drinks, fruit juice, lemonade, coffee and tea with sugar or honey, and yoghurt drinks such as milkshakes. Drinks with sugar substitutes, such as diet soda, were not included.

#### The HS program

The HS program aims to support schools in simulating healthier lifestyles [14] and offers multiple forms of support, such as web-based information, financial support to implement interventions directed towards behaviour change, or compensate additional teaching working hours, training sessions and support from an HS adviser from the regional Public Health Service (Dutch: *GGD*). The requirements to obtain the HS program certificate are specified per health topic (nutrition, physical activity, well-being, smoking, alcohol and drug prevention, as well as relationships and sexuality for secondary schools), and are related to four pillars: health education, school environments (both physical and social), identifying students who need extra attention or referral and healthy school policy [14]. If schools meet these requirements, they can voluntarily apply for a topic certificate. The questionnaire to receive an HS program certificate is self-reported, but the program organisation checks whether the answers are sufficient, and thematic specialists judge more specific elements. When a school acquires a topic certificate, the school also receives the HS program certificate. Additionally, yearly a minimum of 550 schools can obtain more intensive support from the organization HS to implement the HS program for a specific health topic. For secondary schools, this support consisted of advice from an HS adviser (an employee of the Public Health Service), plus 3000–4000 euros financial support and several trainings (since 2017–2018) in how to work with the HS program or on a specific health topic. We included the following characteristics related to the HS program: *HS* (whether a school was a certified HS in the school year 2019–2020); the separate HS topic certificates in 2019–2020 *(nutrition, physical activity, well-being, smoking, alcohol and drug prevention and relationships and sexuality)*; *HS ever* (whether a school was a certified HS at least once since the initiation of the program (2010) [30]); *number of years HS* (the total number of school years a school has or had been a certified HS since the initiation of the program including the school year 2019–2020); *support* (whether a school received more intensive support in 2019–2020); and *total support* (how many school years a school received more intensive support since the school year 2015–2016). If a school obtained the certificate within a certain school year (August 1^st^ was used as cut-off point), the certificate was valid for the corresponding school year and three school years afterwards [14]. HS certification can be considered as a proxy for implementation adherence of the four pillars of the program, but implementation fidelity is not clear and can differ between schools. Schools with the HS program certificate in 2019–2020 will be referred to as Healthy Schools in the remainder of this study.

#### General school- and school population characteristics

The following general school- and school population characteristics, that were obtained from the NCO dataset, were included in the study: *urbanicity* of the school area (low (< 1000 addresses/km^2^), medium (1000–1500 addresses/km^2^) and high (≥ 1500 addresses/km^2^)); *school size* (number of adolescents); *school type* (public, independent non-denominational, Catholic, Protestant or collaboration/other); *poverty level* (percentage of the school population from high-poverty areas); *high parental educational attainment* (the proportion of the school population with at least one parent with high educational attainment); and *migration background* (proportion of the school population with a migration background). Urbanicity was initially measured in five categories and we combined the two highest and the two lowest categories, for high and low urbanicity. Whether an area is classified as a high poverty-area is determined for every postal code area separately. High poverty-areas are classified based on the proportion of very low household incomes, the proportion of households receiving benefit, and the proportion of households with a breadwinner with a non-western migration background. The highest educational attainment of the parents was measured by multiple categories, and we categorised being graduated from higher vocational education or university education as high educated, according to the *Standaard Onderwijsindeling* (SOI) of Statistics Netherlands [31]. Furthermore, the standard survey of the Youth Health Monitor did not measure any variables regarding migration background or highest educational attainment of the parents. This information was therefore included as a school-level estimate obtained from NCO data of secondary school adolescents in their final year. We used data from the school year 2018–2019, since data from 2019–2020 were not included in the NCO dataset from which we obtained the school-level estimate.

Additionally, the following characteristics of the respondents were included in the study: *age* (younger than 14, 14–15, and 16 years and above), *grade (*8^th^ or 10^th^ grade)*, educational track, financial difficulties* at home (yes/no), *psychosocial health*, *happiness*, *truancy,* and *school experience*. Age was truncated in the survey, but the categories ranged from about 12 to 18 years. The educational track of the adolescent was categorised as vmbo-bb/kb, vmbo-gl/tl, havo and vwo. Where an adolescent followed two different tracks, e.g. havo/vwo, we categorised it as the lowest track, since adolescents in the Netherlands are more likely to transition to the lower track later on in their school career [32]. Where an adolescent followed three tracks, this was categorised as the middle one, and if all four tracks were indicated, we categorised this as havo. Where the educational track was missing, we determined this based on the educational structure of the school when possible. Psychosocial health was assessed using the Strengths and Difficulties Questionnaire (SDQ) [33] with total scores ranging from 0 to 40. Scores higher than 12 were specified as borderline/abnormal, based on the guidelines of the Netherlands Organisation for Applied Scientific Research (TNO) [34]. Happiness was measured with a five smileys question ranging from very unhappy to very happy which, for our study, was coded as happy when scoring either happy or very happy. Truancy was measured as skipping school in the four school weeks prior to the assessment. School experience was assessed using a 5-point scale, ranging from ‘very nice’ to ‘horrible’. We indicated whether an adolescent had a positive experience by combining the two highest categories, whether the adolescent had a negative school experience by combining the two lowest options and whether the school experience was average with the middle category. Except for truancy and school experience, all questions were equal in all surveys across all Public Health Services. The questions related to truancy and school experience were included in the survey of only four and five Public Health Services, respectively.

### Statistical analyses

We performed multilevel analyses [35] using the 4.1.3 version of R [36]. Missing data were dealt with through multiple imputation with the mice package [37] using predictive mean matching and (polytomous) logistic regression. Our imputation model consisted of ten imputations and twenty iterations to impute missing values. Truancy, school experience and the SDQ score had the largest amount of missing values (< 30%). All other variables were missing for less than three percent of the adolescents. All variables were included in the imputation model except the HS indicator and whether a school had been a certified HS since its initiation since these can be directly derived from the topic certificates and the number of years a schools has or had been a certified HS. We used the gender of the adolescent and the Public Health Service as auxiliary variables. Auxiliary variables are variables that are related to the variable with missing data and can therefore improve the imputations [38]. Additionally, the five items related to the subscale pro-social behaviour of the SDQ were also added as auxiliary variables. The total SDQ score was calculated during the imputation process by adding up the scores of the twenty SDQ items using passive imputation [39]. We categorised the SDQ score after the imputation to have more information in the imputation model. Lastly, to take into account the differences in dietary intake between schools (i.e. school-level variation), the estimated variance at the school-level for both the consumption of water and SSBs were also included as auxiliary variables.

Our analysis model consisted of two levels: the adolescents and the schools they were nested in. First, we tested the null model with a random intercept to examine the variation between schools regarding the daily consumption of water and SSBs. Based on the results of these models, we calculated the intraclass correlation coefficient (ICC) using the following formula:

$${ICC}_{school}= \frac{{\sigma }_{school}^{2}}{{\sigma }_{school}^{2}+\varepsilon },$$

where ${\sigma }_{school}^{2}$ displays the estimated variance at the school-level and $\varepsilon$ the residual variance, which was defined as *pi*^*2*^*/3* since we used a binomial logistic model [35]. To examine how much of this variance was explained by general school- and school population characteristics, each variable was added univariately to the null model. When a characteristic explained ≥ 10% of the variance between schools, we defined this as meaningful [40, 41]. The variables that explained at least 10% of the variance between schools were added multivariately in one model to obtain the total amount of explained variation. We also examined whether the number of years a school has or had been a certified HS explained differences between schools, including Healthy Schools only. Next, interactions with the HS program certificate/the nutrition certificate and the general school- and school population characteristics were tested. For the cross-level interactions, we added a random slope for the lower level unless this was not possible due to convergence and/or singularity problems [42]. Significant (i.e., *p*-value < 0.05) interaction effects were examined for relevance by inspecting the effect sizes, expressed as odds ratios. All analyses based on multiple imputation were compared to complete case analyses.

## Results

### Descriptive analyses

Table 1 presents the descriptive statistics of secondary schools, separately for schools with and without the HS program certificate in school year 2019–2020, as a proxy for implementation adherence. The flowchart in Additional file 1 presents the number of adolescents included in the analyses and Additional file 2 presents the included number of schools per Public Health Service. In total, 51,901 adolescents in 191 secondary schools were included in the analyses, of which 60 schools had the HS program certificate in 2019–2020 and 8 non-certified schools had been a certified HS before the school year 2019–2020. Of the topic certificates, nutrition was the most common: 55.0% of the Healthy Schools had this certificate. The Healthy Schools had on average been a certified HS for 3.9 years and the non-certified schools for 0.2 years (which is explained by the fact that 8 schools were a certified HS in the past). Regarding the Healthy Schools, 56.7% were located in an area with high urbanicity and 44.3% of the non-certified schools were located in high urbanicity areas. For both groups, most schools were Catholic. The proportion of the school population with at least one high educated parent was not significantly different between Healthy Schools and non-certified schools (0.6 vs. 0.5) (*p* = 0.28). On average, descriptive results show that the most common educational tracks of the adolescents were havo (27.0%) and vmbo-gl/tl (26.8%) in Healthy Schools and vmbo-gl/tl (31.0%) and vmbo-bb/kb (26.9%) in non-certified schools. There were significant differences between (adolescents of) Healthy Schools and non-certified schools for all included characteristics related to the HS program, urbanicity, age, grade, financial difficulties, school experience, and truancy. Irrespective of going to a certified or non-certified school, more than 26% of the adolescents did not drink water daily and over 31% consumed SSBs daily. Of the adolescents that consumed SSBs daily, 64.0% also consumed water daily. The average number of respondents per school was 272.

**Table 1:** Descriptive statistics of sample of secondary schools separately for certified Healthy Schools and non-certified schools

	**Consumption of water and SSBs (*N* = 191)**	
	**Healthy Schools (*N* = 60**^**a**^**)**	**Non-certified schools (*N* = 131**^**a**^**)**
Adolescents (N)	17,698	34,203
Water (daily) (%)	73.2^b^	73.6^c^
SSBs (daily) (%)	31.7^b^	32.5^c^
**Characteristics related to the Healthy School program**		
*Healthy School topic certificates*		
Nutrition (yes)* (%)	55.0	0
Physical activity (yes)* (%)	46.7	0
Well-being (yes)* (%)	16.7	0
Smoking, alcohol and drug prevention (yes)* (%)	16.7	0
Relationships and sexuality (yes)* (%)	10.0	0
Healthy School ever (yes)* (%)	100	6.1
No. of years Healthy School* (Mean (SD))	3.9 (1.1)	0.2 (1.0)
Support* ^d^ (yes) (%)	28.3	3.1
Total support* ^d^ (no. of years) (Mean (SD))	1.1 (1.1)	0.2 (0.5)
**General school- and school population characteristics**		
Urbanicity (%)		
High	56.7	44.3
Medium*	8.3	21.4
Low	35.0	34.4
School size (no. of students) (Mean (SD))	927 (513)	813 (475)
School type (%)		
Public	23.3	23.7
Independent non-denominational	13.3	11.5
Catholic	28.3	30.5
Protestant	21.7	16.0
Collaboration/other	13.3	18.3
Poverty level (%) (Mean (SD))	7.3 (9.9)	7.6 (11.7)
Proportion high parental educational attainment ^e^ (Mean (SD))	0.6 (0.2)	0.5 (0.2)
Proportion migration background ^e^ (Mean (SD))	0.2 (0.1)	0.2 (0.1)
**Respondents described at the school-level** (Mean (SD))^f^		
Age		
Percentage younger than 14 years*	43.6 (13.1)	38.0 (16.2)
Percentage 14–15 years	42.5 (8.6)	43.9 (10.4)
Percentage 16 years and above*	13.9 (7.3)	18.1 (10.5)
Grade		
Percentage grade 8*	55.5 (15.2)	49.4 (19.5)
Percentage grade 10*	44.5 (15.2)	50.6 (19.5)
Educational track ^g^		
Percentage vwo	23.5 (27.6)	19.5 (27.2)
Percentage havo	27.0 (25.0)	22.6 (25.5)
Percentage vmbo-gl/tl	26.8 (23.7)	31.0 (27.5)
Percentage vmbo-bb/kb	22.7 (29.1)	26.9 (33.2)
Percentage financial difficulties* (no)	95.9 (2.0)	95.1 (2.8)
Percentage psychosocial health (normal)	71.1 (6.6)	69.4 (6.4)
Percentage happiness (yes)	85.6 (3.7)	84.8 (4.9)
Percentage truancy* ^h^ (yes)	10.1 (4.7)	12.2 (5.4)
School experience ^i^		
Percentage good	53.6 (7.1)	52.3 (9.4)
Percentage average	36.5 (5.7)	36.4 (7.8)
Percentage bad*	9.8 (2.8)	11.3 (4.6)

*No* number, *SD* Standard deviation, *SSBs* sugar-sweetened beverages

^a^ The number of schools with data unless otherwise stated

^b^ Descriptive statistics are presented on the individual-level for the outcomes. Data were missing from 410 respondents for water consumption and from 412 respondents for SSB consumption

^c^ Data were missing from 698 respondents for water consumption and from 713 respondents for SSB consumption

^d^ Data were available from 127 non-certified schools

^e^ Data were available from 58 Healthy Schools and 124 non-certified schools

^f^ Measured on individual-level, but descriptive statistics are reported at the school-level

^g^ The four vmbo-tracks (vmbo-bb/vmbo-kb, vmbo-gl/vmbo-tl) offer different types of education, vmbo-bb and vmbo-kb are more practically oriented and vmbo-gl and vmbo-tl more theoretically oriented [29]

^h^ Data were available from 39 Healthy Schools and 93 non-certified schools

^i^ Data were available from 46 Healthy Schools and 109 non-certified schools

^*^ = Significantly (*p* < 0.05) different between Healthy Schools and non-certified schools

### Differences in the daily consumption of water and SSBs

Table 2 presents the results of multilevel analyses for the consumption of water and SSBs separately. For water consumption, 4.53% of the difference was explained by differences at the school-level. None of the included characteristics related to the HS program explained variation between the schools with ≥ 10%, but three other characteristics did, i.e. high parental educational attainment, the educational track, and urbanicity. Together (multivariately), these characteristics explained 3.59% (4.53%-0.94%) of the variation between schools. The daily consumption of water was lower among adolescents in schools where less than 50% of the school population had at least one high educated parent (66.1% vs. 76.9%). The higher the educational track of the adolescents, the higher the percentage of adolescents that consumed water daily (vwo = 81.2%, havo = 77.0%, vmbo-gl/tl = 70.6%, vmbo-bb/kb = 63.1%). The same applied for urbanicity (high = 76.0%, medium = 72.8%, low = 69.8%). The variation between schools within Healthy Schools was 4.67%, but the number of years a school has or had been a certified HS did not explain the variation with ≥ 10%. There was also no significant association between the number of years a school has or had been a certified HS and the daily consumption of water within this subgroup. For SSB consumption, 2.33% of the difference was explained by differences at the school-level, when including all schools. HS certification did not explain variation between the schools with ≥ 10%, but three characteristics did, i.e. high parental educational attainment, the educational track of the adolescents, and the school type. The daily consumption of SSBs was higher among adolescents in schools where less than 50% of the school population had at least one high educated parent (35.6% vs. 30.6%). The higher the educational track of the adolescents, the lower the percentage of adolescents that consumed SSBs every day (vwo = 27.7%, havo = 31.3%, vmbo-gl/tl = 33.8%, vmbo-bb/kb = 36.7%). The consumption of SSBs was highest among adolescents in Protestant schools and lowest among adolescents in public schools (36.1% vs. 30.8%). Due to convergence problems, that occurred when multiple variables were included in the logistic model simultaneously, it was not possible to calculate how much variation these characteristics explained multivariately. High parental educational attainment explained most variance between schools, namely 0.91% (2.33%-1.42%). The variation between schools within Healthy Schools was 1.64%, but the number of years a school has or had been a certified HS did not explain the variation with ≥ 10%. There was also no significant association between the number of years a school has or had been a certified HS and the daily consumption of SSBs within this subgroup. Table 3 shows that there were no significant (*p* < 0.05) associations between included characteristics related to the HS program and the daily consumption of water and SSBs. Tables S1 and S2 in Additional file 3 show that there were also no significant interaction effects with the HS program certificate or the nutrition certificate and the general school- and school population characteristics on the daily consumption of water and SSBs. The results of the complete case analyses led to the same conclusions as our main analyses (data not shown).

**Table 2:** Multilevel intraclass correlations in secondary schools for the daily consumption of water and SSBs

	**Water (*N* = 191**^**a**^**) ICC (%)**	**SSBs (*N* = 191 **^**a**^**) ICC (%)**
0 model	4.53	2.33
**Characteristics related to the Healthy School program**		
Healthy School	4.53	2.32
*Healthy School topic certificates*		
Nutrition	4.50	2.33
Physical activity	4.51	2.32
Well-being	4.49	2.33
Smoking, alcohol and drug prevention	4.53	2.32
Relationships and sexuality	4.52	2.32
Healthy School ever	4.53	2.31
Number of years Healthy School	4.53	2.30
Support	4.52	2.33
Total support	4.45	2.33
**General school- and school population characteristics**		
Urbanicity	3.77^d^	2.11
School type	4.47	2.07 ^d^
Poverty level	4.53	2.33
High parental educational attainment	1.29 ^d^	1.42 ^d^
Migration background	4.51	2.30
Age^b^	4.62	2.20
Grade^b^	4.58	2.30
Educational track^b^	1.50 ^d^	1.70 ^d^
Financial difficulties^b^	4.48	2.32
Psychosocial health^b^	4.36	2.28
Happiness^b^	4.56	2.33
Truancy^b^	4.50	2.29
School experience^b^	4.38	2.24
**All significant variables**^**c**^	0.94 ^d^	-

Analyses with school size were not possible due to convergence/singularity warnings. N (adolescents) was 51,901 for all models. *ICC* intraclass correlation, *SSBs* sugar-sweetened beverages,—Analysis was not possible due to convergence/singularity warnings

^a^*N* = Number of schools

^b^ Characteristics of the respondents, measured on an individual-level

^c^ Multivariate analysis including all variables that decreased the ICC by ≥ 10% after inclusion of the variable

^d^ = ICC decreased by ≥ 10% after inclusion of the variable(s)

**Table 3:** Association between Healthy School and the daily consumption of water and SSBs in secondary schools

	**Water (*N* = 191 **^**a**^**)**	**SSBs (*N* = 191**^**a**^**)**
	**OR (95% CI)**	**OR (95% CI)**
*Model 1*		
Intercept	2.71 (2.51–2.91)*	0.48 (0.45–0.51)*
Healthy School	0.99 (0.86–1.12)	0.97 (0.88–1.07)
*Model 2*		
Intercept	2.74 (2.56–2.93)*	0.47 (0.45–0.50)*
Nutrition certificate	0.91 (0.78–1.07)	1.00 (0.89–1.13)
*Model 3*		
Intercept	2.69 (2.49–2.90)*	0.48 (0.46–0.51)*
Healthy School ever	1.00 (0.88–1.14)	0.95 (0.87–1.04)
*Model 4*		
Intercept	2.68 (2.48–2.89)*	0.48 (0.46–0.51)*
Number of years Healthy School	1.00 (0.97–1.04)	0.98 (0.96–1.01)

Reference group = Does not consume water/sugar-sweetened beverages daily. N (adolescents) was 51,901 for all models. *CI* confidence interval, *OR* odds ratio, *SSBs* sugar-sweetened beverages

^a^*N* = Number of schools

^*^ = Significant (*p* < 0.05)

## Discussion

The aim of this study was to examine to what extent differences in the daily consumption of water and SSBs between secondary schools in the Netherlands could be explained by differences between schools regarding HS certification, general school characteristics, and the school population. We also examined whether these general school- and school population characteristics moderated the association between HS certification and the daily consumption of water and SSBs. We found that 4.53% of the total variation in the daily consumption of water and 2.33% of the total variation in the daily consumption of SSBs was accounted for by differences between schools. These low percentages indicate that school-level differences in general do not matter substantially for the daily consumption of water and SSB. Since the focus of this study is the influence of HS certification, general school- and school population characteristics on individual outcomes, we did explore the small variation at the school-level in further analyses.

We compared the characteristics of our study population on the school-level, i.e. the poverty level and the proportion of pupils with a migration background, to the characteristics of all adolescents in publicly funded secondary schools in educational tracks vmbo, havo and/or vwo of the same school year, using the NCO dataset. Schools in our study had on average a lower poverty level (i.e. relatively fewer adolescents from high-poverty areas), and a lower proportion of pupils with a migration background. For both the consumption of water and SSBs, high parental educational attainment explained most variance between schools. This finding is in line with literature findings that reported evidence that parental education [43–46], and school-level socioeconomic status (SES) [47] are associated with the consumption of water and/or SSBs. Our results showed that adolescents in schools where less than half of the school population had at least one high educated parent demonstrated unhealthier behaviours regarding our outcomes, which is in line with international research about socioeconomic health inequalities [23]. We also found that relatively more adolescents in schools in high urbanicity areas consumed water daily compared to adolescents in schools in low- and medium urbanicity areas and that relatively more adolescents in Protestant schools consumed SSBs daily compared to adolescents in public schools. Since high parental educational attainment explained most variance between schools and high educated people live more often in high urbanicity areas [48] and are less often religious [49], this might explain why differences between schools regarding water and SSB consumption were also explained by urbanicity and school type. Besides these characteristics, the adolescent’s educational track partly explained the variance between schools as well, which is also in line with results of the Youth Health Monitor of 2019 throughout the Netherlands [15] and with socioeconomic health inequalities starting at a young age [23].

The variation between schools in the daily consumption of water and SSBs was not explained by HS certification. We also found no significant association between the HS program certificate or the nutrition certificate and the daily consumption of water and SSBs. Since the variation between schools was small and became even smaller after we took into account different general school- and school population characteristics, little variation remained to be explained by HS certification. Nevertheless, if adolescents in certified Healthy Schools had significantly healthier behaviours regarding the daily consumption of water and SSBs in comparison to non-certified schools, we probably would have found larger variation between schools. An explanation for these findings might be that the HS certification in itself may not be a good proxy measurement for implementation of the HS program. It is plausible that some non-certified schools already implemented the HS program, but did not (yet) have a certificate. Non-certified schools may also have implemented other effective health-promoting programs or interventions [50]. Moreover, some criteria for certification are less strict. For example, one of the criteria for the nutrition certificate is the implementation of the HS Canteen intervention [16]. This intervention still allows for having up to 40% of products that can be freely chosen, such as soda, and the other products should be better options, but these can be diet soda or a small bag of crisps [51, 52]. Although a certified HS also has a water tap outside the toilets to stimulate water consumption [16], previous studies showed that adolescents’ consumption of SSBs is probably higher if these drinks are available in the direct school environment [53, 54]. Stricter criteria might enhance the differentiation between certified Healthy Schools and non-certified schools on our outcomes, but they might also induce more effect. For example, by banning the sale of soda or even all SSBs in schools, in addition to adding water taps, as indicated in previous research to be an effective policy [55]. This way, drinking water becomes the social school norm and should not require additional effort to maintain this behaviour [56].

As stated, variation in daily consumption of water and SSBs between schools was small, indicating that the potential impact of the school on the consumption of water and SSBs might be limited. We were not able to include characteristics of the home environment in our study, but previous literature findings highlighted the important role of the availability of SSBs at home, parent modelling, and parental attitudes [24, 25, 57–59] and showed that the majority of SSB consumption takes place at home [60, 61]. The HS program focusses on the involvement of parents, for example by involving them in the school’s policymaking regarding nutrition, by informing parents about the school’s policy and its educational activities related to nutrition, as well as enabling parents to ask questions regarding nutrition [16]. However, since most of these activities and offers are not compulsory for parents, it is unclear how many of them take advantage of it. Given the limited role of schools in water and SSB consumption, our results suggest the potential importance of focusing on the involvement of the home environment even more to reduce the consumption of SSBs both at home and at school. If this implied important condition is met, the currently untapped potential for SHP may be highest in schools that provide the educational tracks vmbo-bb/kb and vmbo-gl/tl and in schools with relatively few adolescents with parents with high educational attainment, since these groups demonstrated less healthy behaviours.

### Strengths and limitations

We included a large number of secondary schools in our analyses and were therefore able to contribute to the existing literature regarding the association between SHP and the consumption of SSBs and water. By combining data from seven Public Health Services across the country, we obtained a good coverage of the Dutch adolescents in secondary schools. Furthermore, the surveys were filled out anonymously, which might have reduced social desirability bias [62]. On the one hand, the large amount of data is a strength of our study, but on the other hand, the fact that we were fully dependent on registration data was also a limitation, due to several reasons: Firstly, as previously mentioned, HS certification might not be a good proxy for the implementation of the HS program and a measure of implementation may be more informative and a more accurate description of the true situation. Additionally, the HS system updates the register in cases where schools merge or split. This could have caused some information bias. However, the data used mostly concerned the school year 2019–2020 and were retrieved in the beginning of 2021. Therefore, we assume that the information bias was limited. Secondly, to obtain a school-level estimate for migration background and high parental attainment of the parents, we used NCO data of adolescents in their final year and assumed their characteristics were representative for the school population, since this information was not obtained in the standard survey of the Youth Health Monitor. This might have caused some information bias.Thirdly, we hypothesised that the impact of HS certification could differ due to different general school- and school population characteristics, but we did not find any evidence to support this hypothesis. Nevertheless, we were limited in the characteristics that could be included in the study. Therefore, we could not include other potentially important moderators, such as the involvement of parents and implementation fidelity. This study is part of a larger evaluation study, and future studies within this project are needed to examine whether SHP can be effective and if so, under which level of implementation and school conditions. Additionally, we were not able to estimate a random slope in all analyses with cross-level interaction terms. However, the impact should be negligible, since the omission of a random slope increases the probability of a type 1 error [42], but none of the *p*-values were significant. Furthermore, the cross-sectional design restricted us to observational conclusions and hampered the examination of causality. Future research should therefore use quasi-experimental designs including a pre- and post-measurement related to the level of implementation [63]. Lastly, there were also some significant differences in characteristics between the (adolescents) of Healthy Schools and non-certified schools, e.g. for age and grade. However, age and grade did not explain variation at the school-level and we also did not find a significant interaction effect with the HS program certificate.

## Conclusions

We found little variation between secondary schools regarding the daily consumption of water and SSBs. Therefore, we conclude that neither school-level characteristics in general nor HS certification matter substantially for the daily consumption of water and SSBs. Nevertheless, our results provide an indication that the untapped potential for SHP may be highest in schools with mostly lower educated parents and adolescents with respect to daily water and SSB consumption, since these groups demonstrated less healthy behaviours. Future studies should examine whether SHP in general and the HS program more specifically are effective under certain conditions, such as a high level of implementation, stricter certificate requirements, or involvement of the home environment. Further research should also examine the impact of the HS program in primary education and secondary vocational education, since the program is also implemented in these schools.

*Results based on calculations by Maastricht University using non-public microdata from Statistics Netherlands. Under certain conditions, these microdata are accessible for statistical and scientific research. For further information: microdata@cbs.nl*. *This research was conducted in part using ODISSEI, the Open Data Infrastructure for Social Science and Economic Innovations (*https://ror.org/03m8v6t10*).*

## Supplementary Information

**Additional file 1.** Flowchart.

**Additional file 2.** Total number of schools in the analyses and in the Netherlands. Description of data: One table that presents the number of adolescents and schools included in the study, compared to the total number of schools and adolescents in the included public health regions and in the Netherlands.

**Additional file 3.** Possible moderators of Healthy School on the daily consumption of water/SSBs in secondary schools.

## Data Availability

The data that support the findings of this study are available from the Association of Regional Public Health Services (*GGD GHOR*), Statistics Netherlands and Healthy School. However, restrictions apply to the availability of these data, which were used under licence for the current study, and so are not publicly available. (Micro)data from Statistics Netherlands, the Health Monitor Youth 2019 and Healthy School are however accessible for statistical and scientific research under criteria defined by the organisations [64–66]. To request the data from this study, you can contact monitorgezondheid@ggdghor.nl (GGD GHOR), microdata@cbs.nl (Statistics Netherlands) and/or info@gezondeschool.nl (Healthy School).

## Abbreviations

CI: Confidence interval
DUO: Dienst Uitvoering Onderwijs
GGD: Regional Public Health Service
GGD GHOR: The Association of Regional Public Health Services
Havo: Senior general secondary education
HPS: Health Promoting Schools
HS: Healthy School
ICC: Intraclass correlation
N/no: Number
NCO: The Netherlands Cohort Study on Education
NRO: The Netherlands Initiative for Education Research
Nu: Nutrition certificate
OR: Odds ratio
Ref: Reference group
SD: Standard deviation
SDQ: Strengths and Difficulties Questionnaire
SES: Socioeconomic status
SHP: School health promotion
SSBs: Sugar-sweetened beverages
TNO: The Netherlands Organisation for Applied Scientific Research
Vmbo: Pre-vocational secondary education
Vwo: Pre-university education
WHO: World Health Organization
