# Supplementary material for: School health promotion and the consumption of water and sugar-sweetened beverages in secondary schools: a cross-sectional multilevel study
Source: BMC Public Health. 2023 Jul 5;23:1296. doi: 10.1186/s12889-023-16123-7 (PMC10324187; doi:10.1186/s12889-023-16123-7)

Additional file 1 Flowchart

File name: Additional file 1

File format: .pdf

Title of data: Flowchart

Description of data: Flowchart of respondents included in the study

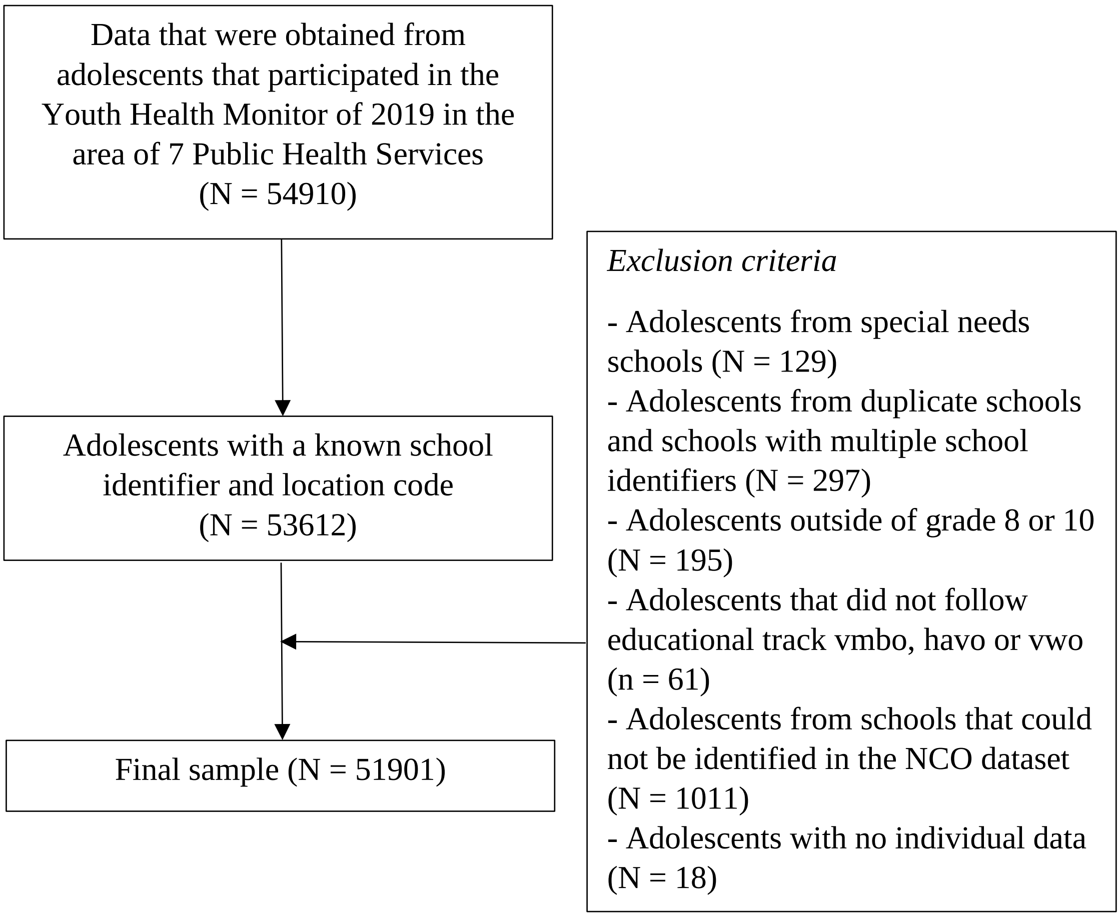

Supplement: Supplementary file 1 — Additional file 1. Flowchart. [file 12889_2023_16123_MOESM1_ESM.pdf]
