# Supplementary material for: School health promotion and the consumption of water and sugar-sweetened beverages in secondary schools: a cross-sectional multilevel study
Source: BMC Public Health. 2023 Jul 5;23:1296. doi: 10.1186/s12889-023-16123-7 (PMC10324187; doi:10.1186/s12889-023-16123-7)
Supplement: Supplementary file 2 — Additional file 2. Total number of schools in the analyses and in the Netherlands. Description of data: One table that presents the number of adolescents and schools included in the study, compared to the total number of schools and adolescents in the included public health regions and in the Netherlands. [file 12889_2023_16123_MOESM2_ESM.pdf]

Additional file 2 Participation rate

File name: Additional file 2

File format: .pdf

Title of data: Total number of schools in the analyses and in the Netherlands.

Description of data: One table that presents the number of adolescents and schools included in the study, compared to the total number of schools and adolescents in the included public health regions and in the Netherlands.

Table S1: Total number of secondary schools in the analyses and in the Netherlands

Public Health Service region	*Schools in region* (N)*	*Schools in analyses (N)*	*% of the total number of schools in the region*
GGD Gelderland-Midden	56	11	19.6%
GGD Gelderland-Zuid	48	31	64.6%
GGD Noord- en Oost-Gelderland	72	54	75.0%
GGD Kennemerland	43	23	53.5%
GGD IJsselland	56	25	44.6%
GGD Limburg-Noord	29	21	72.4%
GGD Zuid Limburg	42	26	61.9%
All 7 Public Health Service regions	346	191	55.2%
the Netherlands	1451	191	13.2%
	Adolescents in region (grade 8 and 10) (N)	*Adolescents in analyses (N)*	*Percentage of the total number of adolescents*
the Netherlands	397292[1]**	51901**	13.1%

**Open access data were derived from Dienst Uitvoering Onderwijs (DUO) [2] regarding school year 2019-2020. Special needs schools are not included in this number. **Adolescents in special needs schools and practical education are not included in this number.*

References

1. Centraal Bureau voor de Statistiek: Statline Vo; leerlingen, onderwijssoort in detail, leerjaar. https://opendata.cbs.nl/statline/#/CBS/nl/dataset/80040ned/table?fromstatweb (2022). Accessed 10 Oct 2022.

2. Dienst Uitvoering Onderwijs: Aantal leerlingen.
https://duo.nl/open_onderwijsdata/voortgezet-onderwijs/aantal-leerlingen/aantal-leerlingen.jsp. Accessed 14 Sep 2022.
