# Supplementary material for: School health promotion and the consumption of water and sugar-sweetened beverages in secondary schools: a cross-sectional multilevel study
Source: BMC Public Health. 2023 Jul 5;23:1296. doi: 10.1186/s12889-023-16123-7 (PMC10324187; doi:10.1186/s12889-023-16123-7)
Supplement: Supplementary file 3 — Additional file 3. Possible moderators of Healthy School on the daily consumption of water/SSBs in secondary schools. [file 12889_2023_16123_MOESM3_ESM.pdf]

Additional file 3

File name: Additional file 3

File format: .pdf

Title of data: Possible moderators of Healthy School on the daily consumption of water/SSBs in secondary schools.

Description of data: Two tables presenting the results of the interaction analyses.

Table S1: Possible moderators of Healthy School on the daily consumption of water/SSBs in secondary schools

	**Water (N = 191[1]) OR (95% CI)**	**SSBs (N = 191[1]) OR (95% CI)**
Model 1: Support		
Intercept (no = ref)	2.70 (2.50-2.91)*	0.48 (0.45-0.51)*
Support	1.07 (0.72-1.58)	0.99 (0.71-1.37)
Healthy School	1.02 (0.88-1.19)	0.96 (0.86-1.07)
HS x support	0.83 (0.52-1.31)	1.06 (0.74-1.54)
Model 2: Total support		
Intercept	2.71 (2.50-2.92)*	0.48 (0.46-0.51)*
Total support	1.00 (0.86-1.17)	0.94 (0.84-1.07)
Healthy School	1.13 (0.95-1.34)	0.91 (0.80-1.03)
HS x total support	0.88 (0.74-1.06)	1.11 (0.97-1.28)
Model 3: Urbanicity		
Intercept (high urbanicity = ref)	3.24 (2.93-3.60)*	0.43 (0.39-0.46)*
Medium urbanicity	0.78 (0.65-0.93)*	1.24 (1.08-1.42)*
Low urbanicity	0.70 (0.60-0.82)*	1.23 (1.09-1.39)*
Healthy School	0.94 (0.80-1.11)	1.06 (0.93-1.20)
HS x medium urbanicity	1.28 (0.84-1.94)	0.82 (0.59-1.13)
HS x low urbanicity	0.97 (0.75-1.26)	0.90 (0.73-1.10)
Model 4: Poverty level		
Intercept	2.71 (2.48-2.96)*	0.48 (0.45-0.51)*
Poverty level	1.00 (0.99-1.01)	1.00 (1.00-1.00)
Healthy School	0.97 (0.83-1.14)	0.97 (0.87-1.10)
HS x poverty level	1.00 (0.99-1.01)	1.00 (0.99-1.01)
Model 5: High parental educational attainment		
Intercept	1.06 (0.92-1.22)	0.84 (0.73-0.97)*
High parental educational attainment	5.69 (4.43-7.29)*	0.35 (0.28-0.45)*
Healthy School	0.90 (0.70-1.15)	0.85 (0.66-1.09)
HS x high parental educational attainment	1.07 (0.71-1.63)	1.34 (0.87-2.04)
Model 6: Migration background		
Intercept	2.68 (2.32-3.09)*	0.51 (0.45-0.56)*
Migration background	1.06 (0.51-2.21)	0.72 (0.41-1.25)
Healthy School	0.87 (0.66-1.15)	0.94 (0.77-1.15)
HS x migration background	2.18 (0.46-10.24)	1.18 (0.39-3.57)
Model 7: School type		
Intercept (public = ref)	2.95 (2.53-3.44)*	0.43 (0.39-0.48)*
Independent non-denominational	0.82 (0.63-1.06)	1.11 (0.92-1.35)
Catholic	0.91 (0.75-1.12)	1.08 (0.93-1.24)

Protestant	0.90 (0.71-1.13)	1.44 (1.22-1.70)*
Collaboration/other	0.91 (0.72-1.14)	1.06 (0.90-1.25)
Healthy School	0.96 (0.73-1.25)	1.04 (0.86-1.26)
HS x independent non-denominational	1.35 (0.86-2.10)	0.84 (0.61-1.15)
HS x Catholic	1.06 (0.74-1.51)	0.93 (0.72-1.20)
HS x Protestant	1.03 (0.69-1.53)	0.79 (0.60-1.05)
HS x collaboration/other	0.81 (0.53-1.23)	1.06 (0.79-1.44)
Model 8: Age		
Intercept (younger than 14 years = ref)	2.59 (2.39-2.81)²*	0.42 (0.40-0.45)*
14-15 years	1.09 (1.03-1.16)*	1.18 (1.12-1.24)*
16 years and above	1.04 (0.96-1.13)	1.36 (1.27-1.46)*
Healthy School	0.96 (0.83-1.10)	1.01 (0.90-1.12)
HS x 14-15 years	1.05 (0.95-1.16)	0.96 (0.88-1.05)
HS x 16 years and above	1.10 (0.96-1.27)	0.97 (0.86-1.09)
Model 9: Grade		
Intercept (grade 8 = ref)	2.58 (2.38-2.79)²*	0.42 (0.40-0.45)²*
Grade 10	1.11 (1.04-1.18)*	1.27 (1.20-1.34)*
Healthy School	0.96 (0.83-1.10)	1.00 (0.89-1.11)
HS x grade 10	1.08 (0.97-1.20)	0.98 (0.89-1.08)
Model 10: Educational track		
Intercept (vwo = ref)	4.08 (3.78-4.42)*	0.38 (0.36-0.41)*
Havo	0.81 (0.75-0.88)*	1.19 (1.11-1.28)*
Vmbo-gl/tl	0.60 (0.55-0.65)*	1.33 (1.23-1.44)*
Vmbo-bb/kb	0.46 (0.42-0.51)*	1.46 (1.33-1.60)*
Healthy School	0.99 (0.87-1.13)	1.06 (0.94-1.19)
HS x havo	0.99 (0.87-1.12)	0.93 (0.83-1.04)
HS x vmbo-gl/tl	0.95 (0.83-1.09)	0.93 (0.81-1.06)
HS x vmbo-bb/kb	0.87 (0.75-1.02)	0.89 (0.77-1.03)
Model 11: Financial difficulties		
Intercept (no = ref)	2.75 (2.56-2.96)*	0.48 (0.45-0.50)*
Yes	0.72 (0.64-0.81)*	1.09 (0.98-1.22)
Healthy School	0.98 (0.86-1.12)	0.96 (0.88-1.06)
HS x yes	1.04 (0.85-1.28)	1.18 (0.97-1.43)
Model 12: Psychosocial health		
Intercept (normal = ref)	3.00 (2.77-3.24)²*	0.45 (0.42-0.47)²*
Borderline/abnormal	0.73 (0.69-0.77)*	1.23 (1.16-1.30)*
Healthy School	1.00 (0.87-1.14)	0.98 (0.89-1.08)
HS x borderline/abnormal	0.95 (0.86-1.04)	0.99 (0.90-1.09)
Model 13: Happiness		
Intercept (yes = ref)	2.81 (2.60-3.04)²*	0.48 (0.45-0.51)²*
No	0.80 (0.74-0.86)*	1.00 (0.93-1.07)
Healthy School	1.00 (0.87-1.14)	0.98 (0.88-1.08)
HS x no	0.93 (0.82-1.05)	0.95 (0.84-1.08)
Model 14: Truancy		
Intercept (yes = ref)	2.21 (1.97-2.47)²*	0.59 (0.54-0.64)*
No	1.26 (1.15-1.39)*	0.79 (0.73-0.85)*
Healthy School	0.99 (0.82-1.20)	0.97 (0.84-1.13)
HS x no	0.99 (0.85-1.15)	1.00 (0.88-1.14)
Model 15: School experience		
Intercept (Good = ref)	3.07 (2.84-3.32)*	0.44 (0.41-0.46)*
Average	0.81 (0.76-0.85)*	1.14 (1.07-1.20)*
Bad	0.69 (0.63-0.75)*	1.47 (1.35-1.59)*
Healthy School	0.99 (0.86-1.14)	1.00 (0.90-1.11)
HS x average	0.99 (0.90-1.09)	0.95 (0.87-1.05)
HS x bad	0.95 (0.83-1.10)	0.89 (0.78-1.03)

Note: ¹ N = Number of schools. ² = Random slope for the lowest level was added to the model. Analyses with school size were not possible due to convergence/singularity warnings. Reference group = Does not consume water/sugar-sweetened beverages daily. Adolescents (N) = 51901. CI = confidence interval; HS = Healthy School (Program certificate); OR = odds ratio; Ref = reference group.

Table S2: Possible moderators of the nutrition certificate on the daily consumption of water/SSBs in secondary schools

	**Water (N = 191[1]) OR (95% CI)**	**SSBs (N = 191[1]) OR (95% CI)**
Model 1: Support		
Intercept (no = ref)	2.73 (2.54-2.92)*	0.47 (0.45-0.50)*
Support	1.05 (0.82-1.34)	1.01 (0.83-1.22)
Nutrition certificate	0.97 (0.81-1.17)	1.00 (0.87-1.14)
Nu x support	0.78 (0.52-1.17)	1.02 (0.75-1.38)
Model 2: Total support		
Intercept	2.76 (2.57-2.97)*	0.47 (0.45-0.50)*
Total support	0.97 (0.89-1.06)	1.00 (0.93-1.07)
Nutrition certificate	1.06 (0.84-1.34)	0.97 (0.82-1.16)
Nu x total support	0.89 (0.75-1.07)	1.03 (0.90-1.17)
Model 3: Urbanicity		
Intercept (high urbanicity = ref)	3.26 (2.97-3.57)*	0.43 (0.40-0.46)*
Medium urbanicity	0.79 (0.67-0.93)*	1.22 (1.07-1.38)*
Low urbanicity	0.69 (0.60-0.79)*	1.18 (1.06-1.31)*
Nutrition certificate	0.88 (0.72-1.07)	1.03 (0.89-1.21)
Nu x medium urbanicity	1.36 (0.73-2.54)	0.70 (0.43-1.15)
Nu x low urbanicity	1.03 (0.75-1.39)	1.02 (0.80-1.29)
Model 4: Poverty level		
Intercept	2.73 (2.52-2.96)*	0.47 (0.45-0.50)*
Poverty level	1.00 (0.99-1.01)	1.00 (1.00-1.00)
Nutrition certificate	0.90 (0.73-1.10)	1.02 (0.88-1.19)
Nu x poverty level	1.00 (0.99-1.02)	1.00 (0.99-1.01)
Model 5: High parental educational attainment		
Intercept	1.08 (0.95-1.23)	0.81 (0.71-0.93)*
High parental educational attainment	5.32 (4.27-6.63)*	0.38 (0.30-0.48)*
Nutrition certificate	0.77 (0.58-1.03)	0.90 (0.66-1.21)
Nu x high parental educational attainment	1.51 (0.90-2.54)	1.17 (0.69-2.00)
Model 6: Migration background		
Intercept	2.69 (2.36-3.06)*	0.49 (0.45-0.54)*
Migration background	1.11 (0.57-2.18)	0.80 (0.48-1.36)
Nutrition certificate	0.78 (0.55-1.09)	1.08 (0.84-1.39)
Nu x migration background	3.01 (0.42-21.44)	0.59 (0.14-2.59)
Model 7: Age		
Intercept (younger than 14 years = ref)	2.60 (2.41-2.79)[2]*	0.42 (0.40-0.45)*
14-15 years	1.11 (1.05-1.17)*	1.16 (1.11-1.22)*
16 years and above	1.07 (1.00-1.15)	1.33 (1.25-1.41)*
Nutrition certificate	0.90 (0.76-1.07)	1.00 (0.88-1.14)
Nu x 14-15 years	1.03 (0.91-1.16)	0.99 (0.89-1.10)
Nu x 16 years and above	1.03 (0.88-1.21)	1.07 (0.93-1.22)
Model 8: Grade		
Intercept (grade 8 = ref)	2.58 (2.41-2.77)[2]*	0.42 (0.40-0.45)[2]*
Grade 10	1.14 (1.07-1.20)*	1.25 (1.19-1.32)*
Nutrition certificate	0.91 (0.77-1.07)	1.00 (0.88-1.14)
Nu x grade 10	1.01 (0.89-1.14)	1.01 (0.90-1.13)
Model 9: Educational track		
Intercept (vwo = ref)	4.08 (3.81-4.37)*	0.39 (0.37-0.42)*
Havo	0.81 (0.76-0.87)*	1.16 (1.09-1.24)*
Vmbo-gl/tl	0.60 (0.56-0.65)*	1.27 (1.19-1.37)*
Vmbo-bb/kb	0.45 (0.42-0.49)*	1.42 (1.31-1.54)*
Nutrition certificate	1.00 (0.85-1.17)	1.00 (0.86-1.16)
Nu x havo	0.97 (0.83-1.14)	0.97 (0.85-1.12)
Nu x vmbo-gl/tl	0.92 (0.78-1.09)	1.07 (0.91-1.25)
Nu x vmbo-bb/kb	0.87 (0.72-1.05)	0.94 (0.78-1.12)
Model 10: Financial difficulties		

Intercept (no = ref)	2.78 (2.60-2.97)*	0.47 (0.45-0.50)*
Yes	0.72 (0.65-0.80)*	1.15 (1.04-1.27)*
Nutrition certificate	0.91 (0.78-1.07)	1.00 (0.89-1.13)
Nu x yes	1.06 (0.83-1.34)	1.02 (0.81-1.29)
Model 11: Psychosocial health		
Intercept (normal = ref)	3.02 (2.82-3.24)²*	0.45 (0.42-0.47)²*
Borderline/abnormal	0.73 (0.69-0.77)*	1.22 (1.16-1.28)*
Nutrition certificate	0.94 (0.80-1.11)	0.99 (0.88-1.12)
Nu x borderline/abnormal	0.91 (0.81-1.02)	1.03 (0.92-1.16)
Model 12: Happiness		
Intercept (yes = ref)	2.84 (2.65-3.05)²*	0.47 (0.45-0.50)²*
No	0.80 (0.77-0.85)*	0.98 (0.92-1.05)
Nutrition certificate	0.93 (0.79-1.10)	1.01 (0.89-1.14)
Nu x no	0.89 (0.77-1.03)	0.98 (0.85-1.14)
Model 13: Truancy		
Intercept (yes = ref)	2.27 (2.05-2.53)²*	0.58 (0.53-0.63)*
No	1.24 (1.13-1.35)*	0.80 (0.74-0.86)*
Nutrition certificate	0.84 (0.66-1.08)	1.09 (0.90-1.31)
Nu x no	1.09 (0.89-1.34)	0.92 (0.77-1.09)
Model 14: School experience		
Intercept (good= ref)	3.10 (2.89-3.32)*	0.44 (0.41-0.46)*
Average	0.80 (0.76-0.85)*	1.12 (1.07-1.18)*
Bad	0.69 (0.64-0.75)*	1.42 (1.31-1.53)*
Nutrition certificate	0.93 (0.79-1.10)	1.01 (0.89-1.15)
Nu x average	0.99 (0.87-1.12)	0.97 (0.87-1.09)
Nu x bad	0.90 (0.76-1.07)	1.00 (0.84-1.18)

Note: ¹ N = Number of schools. ² = Random slope for the lowest level was added to the model. Analyses with school size and school type were not possible due to convergence/singularity warnings. Reference group = Does not consume water/sugar-sweetened beverages daily. Adolescents (N) = 51901. CI = confidence interval; Nu = nutrition certificate; OR = odds ratio; Ref = reference group.
